# Using Evolutionary Computation on GPS Position Correction

**DOI:** 10.1155/2014/723736

**Published:** 2014-01-20

**Authors:** Jung Yi Lin

**Affiliations:** Department of Computer Science and Information Engineering, Chien Hsin University of Science and Technology, Jhongli, Taiwan

## Abstract

More and more devices are equipped with global positioning system (GPS). However, those handheld devices with consumer-grade GPS receivers usually have low accuracy in positioning. A position correction algorithm is therefore useful in this case. In this paper, we proposed an evolutionary computation based technique to generate a correction function by two GPS receivers and a known reference location. Locating one GPS receiver on the known location and combining its longitude and latitude information and exact poisoning information, the proposed technique is capable of evolving a correction function by such. The proposed technique can be implemented and executed on handheld devices without hardware reconfiguration. Experiments are conducted to demonstrate performance of the proposed technique. Positioning error could be significantly reduced from the order of 10 m to the order of 1 m.

## 1. Introduction 

Global positioning System, GPS, has been successfully applied in various areas such as navigation, meteorology, military tasks, mapping, tour design, path tracking tools, and more [1]. Recently many mobile devices have been equipped with embedded GPS [2] such as tablet PCs and smart phones. They provide maps to help users not to lose their way or search the shortest route to their destination.

GPS receiver receives satellite signal from some of constellation 24 GPS satellites. Those satellites are controlled by the United States Department of Defense [2]. The position of a GPS receiver, *u*, can be derived by the pseudorange **ρ** of a satellite. Let *s* be a satellite located at (*x*_*s*_, *y*_*s*_, *z*_*s*_); *u* is located at (*x*_*u*_, *y*_*u*_, *z*_*u*_); *ρ* can be evaluated by

(1) ρ=||s−u||+ctr+vρ=(xs−xu)2+(ys−yu)2+(zs−zu)2+ctr+vρ,

where *c* is the speed of light; *t*_*r*_ is the offset of the receiver clock; and *v*_*ρ*_ stands for a random noise that is expected to be zero.

Theoretically, the exact position of *u *(*x*_*u*_, *y*_*u*_, *z*_*u*_) can be determined with given four error-free satellite coordinates. Unfortunately, GPS positional accuracy is affected by many factors [3, 4] such as radio signal corruption, ephemeris error, satellite and receiver clock offset, multipath error, receiver measurement noise, satellite geometry measures, tropospheric delay, and ionospheric delay [5]. In general, due to those noises, GPS position accuracy is degraded to the order of 10 m [6].

Many techniques are proposed to improve GPS position accuracy. A commonly used technique is to use relative positioning [7]. Relative positioning methods, including static, rapid static, pseudokinematic, kinematic, and real-time kinematic [7–9], have proved their ability of improving GPS accuracy. In [7], Berber et al. claimed that pseudokinematic technique produces closest results, which could significantly reduce the error to 2 centimeters.

**Figure 1:**
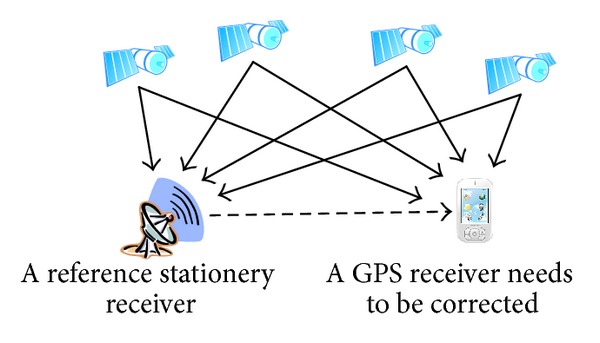
An illustration of dGPS scenario. The precise location of the reference stationary receiver is known. A GPS receiver which requires correction accepts both signals from satellites and the stationary receiver.

Differential correction is an effective method to improve GPS positional accuracy. A GPS receiver with such technique is called dGPS. A typical differential correction requires a reference stationary receiver at a known location [10, 11].  Figure 1 shows a typical scenario of the dGPS environment. The exact location information of reference stationary receiver is known. It receives GPS signals and calculates its position. Under the assumption that close GPS receivers suffer similar noises and after evaluating the difference between the exact known position information and the calculated position information, the reference stationary receiver communicates with roving GPS receivers to correct their position information. dGPS can be used to eliminate affections of ionospheric and tropospheric delay, ephemeris error, and satellite clock error. However, when the error is due to multipath error, or poor satellite measurement geometry, the improvement effectiveness of dGPS technique is relatively low.

**Figure 2:**
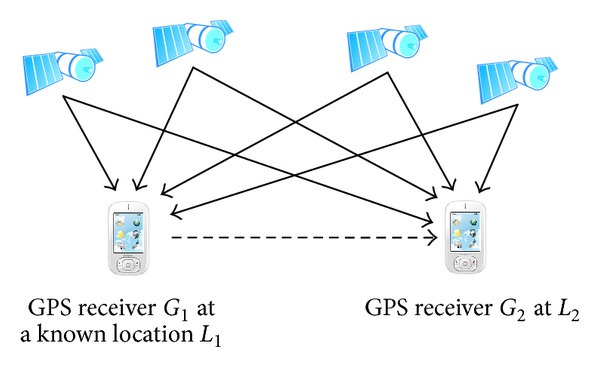
A consumer-grade GPS receiver at a location which has exact known location.

The main drawback of using dGPS technique is that reference stationary receivers are not common in many countries. Fortunately, many accessible places have been precisely measured for their geometry location. If a consumer-grade GPS receiver could be a reference stationary receiver, it is possible to simulate a dGPS environment. Given two GPS receivers, *G*_1_ and *G*_2_, where *G*_1_ is placed on a known location, *L*_1_, the location information obtained by *G*_1_ could be used to correct *G*_2_. Such scenario is shown in Figure 2. In this paper, we will use two consumer-grade GPS receivers to construct the scenario. Instead of using survey-grade GPS receivers, which have high accuracy and have been applied correction techniques, consumer-grade GPS receivers could be more common for most of users.

A navel position correction technique is proposed in this paper. This technique is based on differential correction and genetic programming (GP) [12]. GP will be used to generate a correction function from NMEA information [13] derived from the GPS receiver at the known location and the GPS receiver which needs to be corrected. The receiver which requires to be corrected will apply the function to obtain its corrected location information.

## 2. Layered Architecture Genetic Programming

Genetic programming [12, 14] is a research area of evolutionary computation. It has been proved that GP is capable of finding a solution efficiently. GP, like other techniques in evolutionary computation, generates possible solutions—in this case, correction functions—randomly for the given problem under given constrains. These solutions are called *individuals*. In this paper, individuals are represented as functional expressions. The fitness value which of an individual is used to measure the degree of the individual fitting with the given problem is determined by a predefined fitness function. The set with fixed size of individuals is named a *population*. In order to produce new solutions, genetic operators such as crossover and mutation are applied on selected individuals, called *parents*, to create offspring and mutant. Comparing the fitness degree of those offspring and mutant with parents, which have higher fitness value, will be kept as survived individuals. All survived individuals will replace the original population. A *generation* is finished once the original population is fully replaced. After a number of generations, evolutionary process completes and the individual with highest fitness is regarded as the result [14].

**Figure 3:**
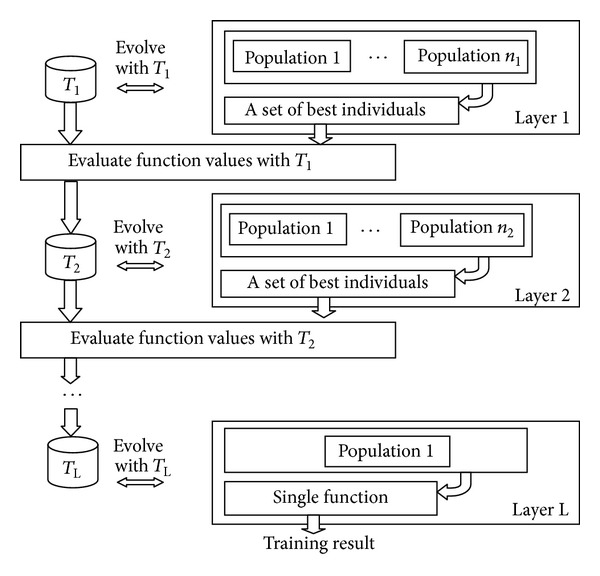
The flowchart of LAGEP.

In this paper, we use the improved version of genetic programming called layered architecture genetic programming, LAGEP [14]. LAGEP is only usable with functional expression individuals. It utilizes the layer architecture to arrange populations. Populations in the same layer evolve independently. Once every population finishes evolutionary progress, the best individual of each population evaluates with its training instances, *T*, to generate a series of numerical results. The number of results is equal to *|T|*. Combining those values, a new training set *T*′ having *|T|* instances could be produced. Supporting that the number of populations in the layer is *n*, *T*′ will be an *n*-dimensional training set. The final layer of LAGEP contains one population only. The individual produced by this population is the evolutionary result [14]. The flowchart of LAGEP is shown in Figure 3.

Training instances are constructed by raw information obtained from two GPS receivers and the known location. GPS receivers are capable of transferring different types of NMEA interpreted sentences [13]. In this work, we used GPGGA to represent position information, as shown in Table 1. The third, fifth, tenth, and twelfth field are symbols that can be harmlessly eliminated. The value of sixth field indicates GPS quality which is fixed. The thirteenth and fourteenth are usable when dGPS is available. The fifteenth is the checksum used to identify correctness of received data. In conclusion, 8 out of 15 fields can be removed. Two GPS receivers construct a 13-feature training instance after eliminating a redundant UTC time feature since those GPS receivers would have identical UTC time. Those features with longitude and latitude of the known location form a 15-feature training instance, as shown in Table 2. The target value is either known latitude or known longitude to which we intent to correct GPS receiver as close as possible.

An individual, idv, is defined as a functional expression composed of variables, operators, and constants:

(2) $$\begin{array}{c} \text{idv}=\left(S_{v},S_{\text{op}},C\right),\\ S_{v}=\left\{X_{i}\;|\;i=1,2,\ldots ,17\right\},\\ S_{\text{op}}=\left\{+,-,\times ,/,\sin,\cos,\log\right\},\\ C=\left\{0,0.1,0.2,0.3,0.4,0.5,0.6,0.7,0.8,0.9,1.0\right\}. \end{array}$$

An individual is a function mapping 17 real value features with constants into single real value, that is, idv: (**R** ^|17|^ ∪ *C*) → **R**, which is supposed to be as close as the target value. The target value is the value what an individual is evolving for. When we attempt to acquire a correction function for latitude, the latitude information will be the target value during this run of the evolutionary process and is the only thing concerned by an individual.

A training instance, *t*, and the training set, *T*, are defined as follows:

(3) $$\begin{array}{c} t=\left(\text{target}\;\text{value},f_{1},f_{2},\ldots ,f_{15}\right),\\ T=\left\{t_{i}\;|\;i=1,2,\ldots ,\left|T\right|\right\}; \end{array}$$

the target value is either known latitude or known longitude. The fitness of an individual is defined by

(4) $$\begin{array}{c} \text{fitness}=\sqrt{\sum_{i=1}^{\left|T\right|}{\left(\text{idv}\left(t_{i}\right)-\text{target value}\right)}^{2}}, \end{array}$$

where idv (*t*_*i*_) stands for the calculated valued of training instance *t*_*i*_ by the individual. Overfitting is a situation that a trained individual highly fits the training set but obtains relatively poor performance for the test set. To avoid the occurrence of such phenomenon, the validation process is applied. An individual having highest score is the output of the population:

(5) $$\begin{array}{c} \text{score}=\text{fitness}+\sqrt{\sum_{i=1}^{\left|V\right|}{\left(\text{idv}\left(t_{i}\right)-\text{target value}\right)}^{2}}, \end{array}$$

where *|V|* is the number of instances in the validation set.

## 3. Experiments

**Figure 4:**
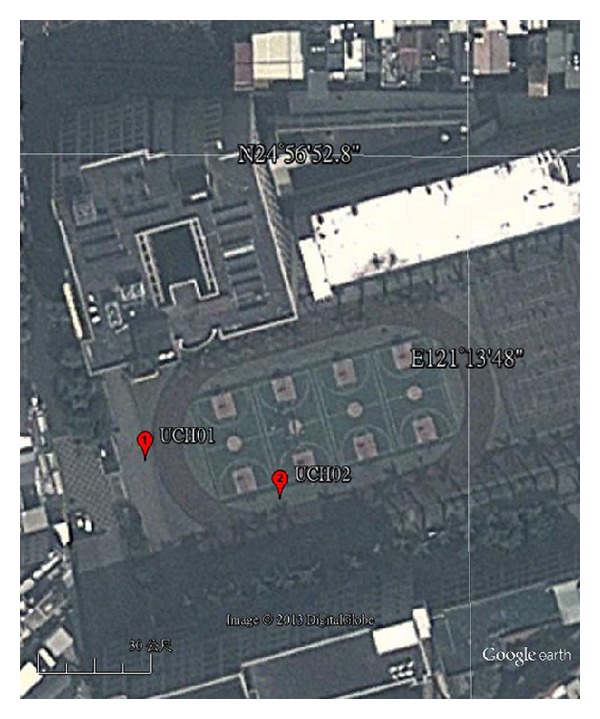
The image of UCH01 and UCH02 (image from Google with a proportional scale of 30 m).

Two public reference positions, UCH01 (24.94728, 121.22916) and UCH02 (24.94719, 121.22951), are provided by Chien Hsin University as shown in Figure 4. Satellite image extracted from Google Earth is shown in Figure 3. Two consumer-grade GPS, HOLUX GPSport 245 [15], are precisely placed on UCH01 and UCH02 for 24 hours to collect position information. After eliminating noisy data, the dataset contains 59,209 instances. We used 19737, 19736, and 19736 instances as training set, validation set, and test set, respectively. To reduce the conversion error in calculating longitude/latitude format, UCH01 and UCH02 are transformed into NMEA format (2456.8368, 12113.7496) and (2456.8314, 12113.7706), respectively:

(6) $$\begin{array}{c} 24.94728:=2400+0.94728\times 60=2456.8368,\\ 121.22916:=12100+0.22916\times 60=12113.7496,\\ 24.94719:=2400+0.94719\times 60=2456.8314,\\ 121.22951:=12100+0.22951\times 60=12113.7706. \end{array}$$

In this paper, we conduct two experiments that use UCH01 and UCH02 to be the reference point and the target position in turns. We performed 5-fold cross-validation for 10 times to demonstrate the average performance. Settings used for GP are shown in Table 3.

**Figure 5:**
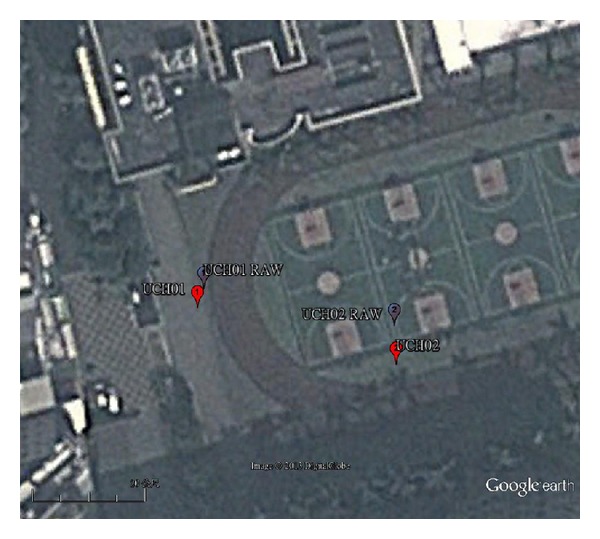
The image of UCH01, UCH02 and the position obtained by GPS receivers (image from Google with a proportional scale of 20 m).

Average distance errors between position information obtained by GPS receivers and the two fixed positions are considerable. Average error is in order of 10 meter, as summarized in Table 4. We also show the image of UCH01 and UCH02 and obtained position information by averaging 59209 instances in Figure 5. Obviously, position information obtained by GPS receivers is unstable and inaccurate.

The training phase of GP is time consuming. Training time records of experiments are summarized in Table 5. It requires about one hour completing one experiment. It seems that the training time is not acceptable in real scenario. However, it is difficult to have that much position information to be training instances in real scenario as well. The training time is affected by the number of training instances. Fewer training instances would greatly cost less training time.

Before showing the experimental results of LAGEP, we demonstrate a simple correction method based on location information obtained by both GPS receivers. Since *G*_1_ is placed right on UCH01, longitude and latitude of *G*_2_ minus the difference between *G*_1_ and UCH01 should be close to UCH02. Denote longitude and latitude information reported by GPS *G*_1_ and *G*_2_ as *G*_1long_, *G*_1la_, *G*_2long_, and G_2la_. The correct longitude and latitude of UCH01 and UCH02 are denoted as UCH01_long_ and UCH01_la_ and UCH02_long_ and UCH02_la_. Location information of *G*_2_, denoted as *G*_2long_′ and *G*_2la_′, is corrected by *G*_1_ using

(7) $$\begin{array}{c} {G_{2\text{long}}}^{\prime }=G_{2\text{long}}-\left(G_{1\text{long}}-{\text{UCH0}1}_{\text{long}}\right),\\ {G_{2\text{la}}}^{\prime }=G_{2\text{la}}-\left(G_{1\text{la}}-{\text{UCH0}1}_{\text{la}}\right). \end{array}$$

*G*_1_ is corrected by *G*_2_ using

(8) $$\begin{array}{c} {G_{1\text{long}}}^{\prime }=G_{1\text{long}}-\left(G_{2\text{long}}-{\text{UCH0}2}_{\text{long}}\right),\\ {G_{1\text{la}}}^{\prime }=G_{1\text{la}}-\left(G_{2\text{la}}-{\text{UCH0}2}_{\text{la}}\right). \end{array}$$

**Figure 6:**
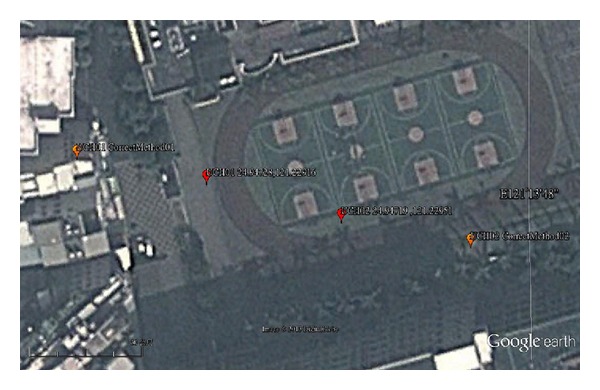
The image of UCH01, UCH02 and the corrected positions using simple correction method (image from Google with a proportional scale of 30 m).

The average error is shown in Tables 6 and 7. The corrected positions are shown in Figure 6. The corrected method seems reasonable but is inaccurate.

**Figure 7:**
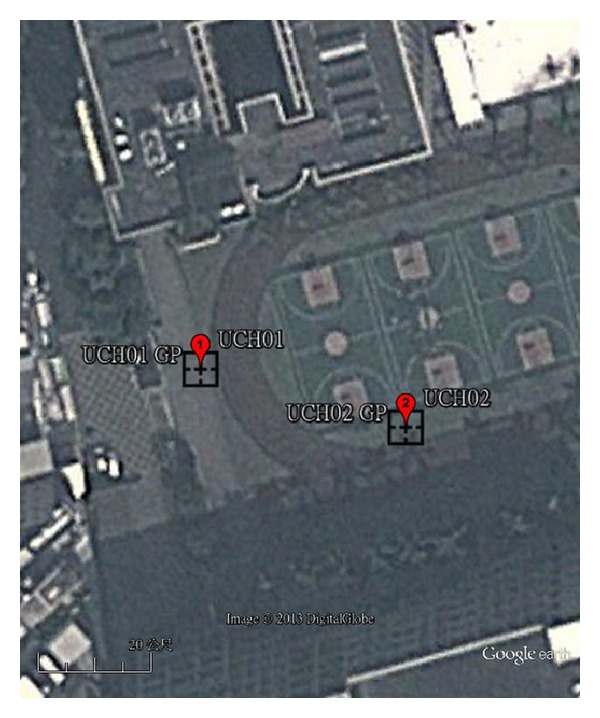
The image of UCH01, UCH02 and the corrected positions using proposed method in black squares (image from Google with a proportional scale of 20 m).

Experiment results of LAGEP on test sets are shown in Table 6. The average corrected position is close to target position with less than 1 meter. It demonstrated that the proposed method achieved significant result in both latitude and longitude. The degree of correction is significant. Standard deviation of those experiments shows that the experimental results are stable. We illustrate the corrected positions in Figure 7. The corrected positions are almost overlapping with UCH01 and UCH02.

## 4. Conclusion

In this paper, we proposed a new GPS position correction technique based on layered genetic programming and the concept of dGPS. Experiments have shown that even when two GPS receivers have high error and noise, the proposed technique is capable of finding correction function to help find accurate position information. The proposed technique could be easily implemented on mobile devices because it does not need to modify or install any hardware component. Our future work will focus on training correction function with time limitation constraints. The training phase stops when given time limitation is reached. Such would be closer to real world.

## Figures and Tables

**Table 1:** Fifteen fields of GPGGA sentence.

Number	Meaning
1	UTC of position
2	Latitude
3*	N or S
4	Longitude
5*	E or W
6*	GPS quality indicator 1: invalid 2: GPS fix 3: dGPS fix
7	Number of satellites in use
8	Horizontal dilution of position
9	Antenna altitude above/below mean sea level (geoid)
10*	Meters (antenna height unit)
11	Geoidal separation
12*	Meters (units of geoidal separation)
13*	Age in seconds since last update from differential reference station
14*	Differential reference station ID
15*	Checksum

*Removed features.

**Table 2:** Features of a training instance.

Number	Meaning
Target value	Known latitude/known longitude
1–7	$GPGGA from GPS receiver 1 as shown in Table 1
8–13	$GPGGA from GPS receiver 2 as shown in Table 1 without the UTC of position
14	Known latitude (where GPS receiver 1 is located)
15	Known longitude (where GPS receiver 1 is located)

**Table 3:** Genetic programming parameters.

Parameter	Value
Number of populations	Layer 1: 4 Layer 2: 2 Layer 3: 1
Population size	Layer 1: 250 for each population Layer 2: 500 for each population Layer 3: 1000 total: 3000
Generations	200
Crossover rate	0.9
Mutation rate	0.1
Operators	+, −, ×, /, sin, cos, natural log
Constants	0.1, 0.2, 0.3,…, 0.9, 1.0, *π*
Depth of an individual	8

**Table 4:** Average error between GPS receivers and reference positions.

	Average error in latitude	Average error in longitude
GPS receiver at UCH01 (2456.8368, 12113.7496)	0.0050057407	0.0208154402
GPS receiver at UCH02 (2456.8314, 12113.7706)	0.0077285210	0.0203315121

**Table 5:** Training time (in second).

Experiments	Target value: 2456.8368	Target value: 12113.7496
Experiment 1	3300.036	3878.202
Experiment 2	2588.838	4169.452
Experiment 3	2965.790	3083.224
Experiment 4	2676.224	3596.150
Experiment 5	2747.012	3520.266
Experiment 6	2364.806	4144.188
Experiment 7	2259.692	3614.860
Experiment 8	2433.826	3692.286
Experiment 9	2425.836	3876.082
Experiment 10	3137.874	3056.296

Average	2689.993	3663.101

Experiments	Target value: 2456.8314	Target value: 12113.7706

Experiment 1	2310.404	4091.588
Experiment 2	2323.656	3270.206
Experiment 3	2271.540	2818.054
Experiment 4	1891.328	4092.478
Experiment 5	1810.772	3855.086
Experiment 6	1856.274	3992.694
Experiment 7	2531.930	3416.518
Experiment 8	2775.832	4032.888
Experiment 9	2767.734	3770.812
Experiment 10	2581.238	3746.426

Average	2312.071	3708.675

**Table 6:** Average error on correct 1.

	Average error in latitude	Average error in longitude
GPS receiver at UCH01	0.0101397676	0.0411469523
GPS receiver at UCH02	0.0101397676	0.0411469523

**Table 7:** Average error of test set.

Experiments	Target value 2456.8368	Target value 12113.7496
Experiment 1	3.60*E* − 11	2.03*E* − 06
Experiment 2	7.98*E* − 08	8.69*E* − 07
Experiment 3	2.74*E* − 08	2.49*E* − 06
Experiment 4	1.27*E* − 06	2.33*E* − 07
Experiment 5	4.61*E* − 06	2.73*E* − 06
Experiment 6	1.09*E* − 09	5.02*E* − 07
Experiment 7	1.90*E* − 07	2.50*E* − 06
Experiment 8	1.71*E* − 06	1.01*E* − 08
Experiment 9	1.46*E* − 08	4.41*E* − 06
Experiment 10	1.84*E* − 07	5.20*E* − 06

Average	8.09*E* − 07	2.10*E* − 06
Standard deviation	1.39*E* − 06	1.66*E* − 06

Experiments	Target value 2456.8314	Target value 12113.7706

Experiment 1	2.50*E* − 11	5.12*E* − 07
Experiment 2	0.00*E* + 00	1.48*E* − 06
Experiment 3	2.00*E* − 12	1.16*E* − 07
Experiment 4	0.00*E* + 00	3.21*E* − 06
Experiment 5	0.00*E* + 00	3.11*E* − 06
Experiment 6	4.49*E* − 09	6.13*E* − 07
Experiment 7	4.00*E* − 12	1.10*E* − 07
Experiment 8	4.00*E* − 12	1.64*E* − 06
Experiment 9	1.00*E* − 12	5.02*E* − 07
Experiment 10	1.00*E* − 12	2.89*E* − 08

Average	4.53*E* − 10	1.13*E* − 06
Standard deviation	1.35*E* − 09	1.14*E* − 06

## References

[1] Frenzel LE (2007). GPS takes a global position in the portable market. *Electronic Design*.

[2] EI-Rabbany A (2002). *Introduction to GPS: the Global Positioning System*.

[3] Yeh TK, Wang CS, Lee CW, Liou YA (2006). Construction and uncertainty evaluation of a calibration system for GPS receivers. *Metrologia*.

[4] Zhang J, Zhang K, Grenfell R, Deakin R (2006). GPS satellite velocity and acceleration determination using the broadcast ephemeris. *Journal of Navigation*.

[5] Klobuchar JA (1996). Ionospheric effects on GPS. *Global PositionIng System: Theory and Applications*.

[6] Arnold LL, Zandbergen PA (2011). Positional accuracy of the Wide Area Augmentation System in consumer-grade GPS units. *Computers and Geosciences*.

[7] Berber M, Ustun A, Yetkin M (2012). Comparison of accuracy of GPS techniques. *Measurement*.

[8] Ghilani CD, Wolf PR (2007). *Elementary Surveying—An Introduction to Geomatics*.

[9] Van Sickle J (2008). *GPS For Land Surveyors*.

[10] Chivers M (2003). *Differential GPS Explained. ArcUser*.

[11] Bolstad P, Jenks A, Berkin J, Horne K, Reading WH (2005). A comparison of autonomous, WAAS, real-time, and post-processed global positioning systems (GPS) accuracies in northern forests. *Northern Journal of Applied Forestry*.

[12] Koza JR (1992). *Genetic Programming: on the Programming of Computers by Means of Natural Selection*.

[13] National Marine Electronics Association (NMEA) http://www.nmea.org/.

[14] Lin J-Y, Ke H-R, Chien B-C, Yang W-P (2007). Designing a classifier by a layered multi-population genetic programming approach. *Pattern Recognition*.

[15] HOLUX Technology Inc http://www.holux.com/JCore/en/products/products_content.jsp?pno=349.

